# A novel method for studying airway hyperresponsiveness in allergic guinea pigs in vivo using the PreciseInhale system for delivery of dry powder aerosols

**DOI:** 10.1007/s13346-018-0490-z

**Published:** 2018-02-21

**Authors:** A. J. Lexmond, S. Keir, W. Terakosolphan, C. P. Page, B. Forbes

**Affiliations:** 1 Institute of Pharmaceutical Science, King’s College London, London, SE1 9NH UK; 2 Sackler Institute of Pulmonary Pharmacology, Institute of Pharmaceutical Science, King’s College London, London, SE1 9NH UK; 3 Present Address: Department of Pharmaceutical Technology and Biopharmacy, Groningen Research Institute of Pharmacy, University of Groningen, 9713 AV Groningen, The Netherlands

**Keywords:** Adenosine, Adenosine 5′-monophosphate, Bronchoconstriction, Asthma, Drug development, Dry powder inhalation

## Abstract

Inhaled adenosine receptor agonists induce bronchoconstriction and inflammation in asthma and are used as bronchial challenge agents for the diagnosis of asthma and in respiratory drug development. Recently developed dry powder aerosols of adenosine have several advantages over nebulised adenosine 5′-monophosphate (AMP) as bronchial challenge agents. However, reverse translation of this bronchial challenge technique to pre-clinical drug development is limited by the difficulty of administering powder aerosols to animals. The aim of the current study was to develop methods for delivering powder aerosols of adenosine receptor agonists to sensitised guinea pigs (as a model of allergic asthma) and evaluate their effect as challenge agents for the measurement of airway responsiveness. The PreciseInhale system delivered micronised AMP and adenosine powders, with mass median aerodynamic diameters of 1.81 and 3.21 μm and deposition fractions of 31 and 48% in the lungs, respectively. Bronchoconstrictor responses in passively sensitised, anaesthetised, spontaneously breathing guinea pigs were compared to responses to nebulised and intravenously administered AMP and adenosine. AMP- and adenosine-induced bronchoconstriction following all routes of administration with the magnitude of response ranking intravenous > dry powder > nebulisation, probably reflecting differences in exposure to the adenosine agonists delivered by the different routes. In conclusion, the PreciseInhale system delivered AMP and adenosine dry powder aerosols accurately into the lungs, suggesting this method can be used to investigate drug effects on airway responsiveness.

## Introduction

Airway hyperresponsiveness (AHR) is present in nearly all patients with asthma and in many patients with chronic obstructive pulmonary disease (COPD) [1]. AHR is an increase in the sensitivity and reactivity of the airways in response to airway exposure to nonspecific stimuli and is commonly measured by means of a bronchial challenge test [2, 3]. The gold standard challenge agent for quantifying AHR is methacholine which acts directly on airway smooth muscle cells [4]. However, use of the indirectly acting stimulus adenosine may provide diagnostic benefits since AHR to adenosine is more mechanistically representative of the disease pathology and airway inflammation than AHR to methacholine [5, 6]. On this basis, it has been argued persuasively that adenosine bronchial challenge testing is a better non-invasive tool for monitoring disease activity and an improved method for assessing the response to anti-inflammatory treatments [7]. Moreover, recent findings suggest that bronchial challenging with adenosine may improve diagnostic discrimination between asthma and COPD [8].

Until recently, the only way to deliver adenosine to the lungs was by means of nebulisation for which solutions of adenosine 5′-monophosphate (AMP) rather than adenosine itself have been used because of AMP’s higher aqueous solubility. Following inhalation, AMP is rapidly hydrolysed to adenosine by 5′-nucleotidase. However, nebulisation of AMP has a number of drawbacks. First, the maximum aerosol droplet AMP concentration (300–400 mg/mL; restricted by the nebuliser solution’s viscosity) does not result in AHR in all patients [8, 9]. Furthermore, nebuliser solution AMP concentrations > 20 mg/mL have been shown to greatly affect aerosol formation, which may have implications for the test outcome, such as a shift in deposition site or disproportional dose increase [10]. Delivery of adenosine as a dry powder aerosol overcomes the issues identified above [11, 12], and bronchial challenge in subjects with asthma has demonstrated that adenosine and AMP appear to induce airway obstruction in a similar manner [13]. Based on these findings, we reasoned that if dry powder adenosine is useful clinically as an inducer of airway obstruction, this should be reverse translated into pre-clinical models to provide more relevant test systems for studying asthma pathophysiology and to aid in the development of novel drug treatments for affecting AHR.

The aim of this study therefore was to develop improved methods to deliver adenosine receptor agonist powder aerosols to sensitised guinea pigs (as a model of allergic asthma) to measure airway responsiveness. In this feasibility/proof-of-concept study, we have investigated whether administration of dry powder aerosols of adenosine agonists can be used to induce airway obstruction in a pre-clinical model of allergic asthma. Our group has previously shown that nebulised AMP and *N*^6^-cyclopentyladenosine (CPA; an adenosine A_1_ receptor agonist) induce airway obstruction in allergic, but not naïve, guinea pigs that were anaesthetised and artificially ventilated [14]. However, the urethane-induced anaesthesia used in these studies can suppress respiration and thus does not allow animals to breathe spontaneously with sufficient tidal volume to permit inhalation of a dry powder aerosol. Therefore, we first needed to develop a new anaesthetic regime to allow aerosols to be inhaled by spontaneously breathing, unconscious guinea pigs. To deliver the dry powder, we utilised a relatively new technique to administer dry powder aerosols to animals, the PreciseInhale system (Inhalation Sciences, Sweden). This system generates a dispersed aerosol cloud approaching the primary particle size of the powder, which is delivered to an individual animal with minimised loss of drug and accurate computer-controlled dose administration [15, 16]. The current paper describes the novel anaesthetic regime, evaluates the use of the PreciseInhale system for delivering adenosine receptor agonist powders to sensitised guinea pigs and compares the resultant bronchial obstruction with that produced by nebulisation and intravenous delivery of the same agents.

## Materials and methods

### Drugs

The following reagents were used in this study: adenosine, adenosine 5′-monophosphate (AMP), ovalbumin (grade V), urethane (all Sigma-Aldrich, UK), propofol, isoflurane (Centaur Services, UK) and the selective adenosine A_1_ receptor agonist *N*^6^-cyclopentyladenosine (CPA) (Tocris, UK).

### Dry powder aerosol preparation

#### Spray drying

Spray-dried powders of adenosine and AMP were prepared following the same procedure used for the adenosine powders that were tested clinically [11]. Aqueous solutions of adenosine or AMP at a concentration of 25 mg/mL were spray dried with a Büchi B290 Mini Spray Drier (Büchi Labortechnik, Switzerland) under the following conditions: compressed nitrogen flow rate 650 L/h, aspirator 100%, solution feed rate 2.5 mL/min, inlet temperature 120 °C, nozzle size 0.7 mm. The outlet temperature was around 73 °C. The adenosine solution was kept at 50 °C during spray drying to prevent precipitation of the solute.

#### Micronisation

Adenosine, AMP and CPA were micronised using an Alpine AS 50 jet mill (Hosakawa, Germany). For adenosine and AMP, a nozzle pressure of 3 bar and a milling pressure of 1 bar were applied. Adenosine was micronised twice using the same settings to obtain a powder with the desired particle size. CPA was micronised by applying a nozzle pressure of 1.5 bar and a milling pressure of 0.5 bar.

#### Primary particle size analysis

The primary particle size distributions (PSDs) of the micronised and spray-dried adenosine receptor agonists were measured with a HELOS BR laser diffraction apparatus (Sympatec, Germany) using a 100-mm (R3) lens and the FREE calculation mode based on the Fraunhofer theory. The powders were dispersed with a RODOS disperser at 3 bar (Sympatec). All measurements were performed in duplicate.

#### Solid-state characterisation

The effects of micronisation and spray drying on the solid-state properties of the adenosine receptor agonists were investigated with differential scanning calorimetry (DSC) and X-ray powder diffraction (XRPD) analysis. Thermal analysis characterising physical transformations was performed with a DSC Q2000 (TA Instruments, Belgium). Samples of 2–5 mg were weighed in open aluminium pans. Samples were preheated at 70 °C for 10 min to remove any adsorbed water and subsequently heated from 0 to 150 or 200 °C (CPA) or 260 °C (adenosine and AMP) at a rate of 20 °C per minute. X-ray diffraction patterns were determined using a D2 Phaser diffractometer (Bruker, the Netherlands) at a scanning rate of 0.004°/min over a 2*θ* range of 5–60° using a 30-kV voltage and a 10-mA current.

#### Dry powder dispersion using the PreciseInhale system

**Fig. 1:**
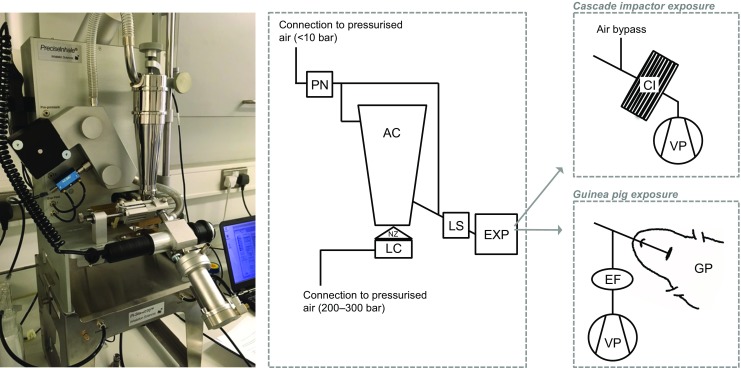
The PreciseInhale system. A powder dose is loaded into the loading chamber, which is then pushed through the nozzle by a high-pressure air jet into the aerosol chamber, where it subsequently settles by opposing force of gravity. It is then released towards the exposure module, for which the flow is created by a vacuum pump. A light-scattering device measures the particle density in the aerosol, which is used to track the emitted dose. The exposure modules used in this study were a Marple cascade impactor and intracheally intubated guinea pigs. Various valves are present in the tubing to allow pressure build-up and release as well as the air to flow in the correct direction. These have been omitted for clarity. A more complete explanation of the PreciseInhale system can be found in reference [17]. AC aerosol chamber, CI cascade impactor, EF end filter, EXP exposure module, GP guinea pig, LC loading chamber, LS light-scattering measuring device, NZ nozzle, PN pneumotachograph, VP vacuum pump

Dry powder aerosols were generated with the PreciseInhale system which has been described in detail previously [17]. Briefly, a powder sample is loaded into the dosing chamber and then vertically ejected by means of a short high-pressure air jet through a nozzle into a cylindrical holding chamber. From here, the aerosol is transferred by airflow to the animal or collected for analysis. The rate of aerosol transfer is controlled by either superimposed over- and/or under-pressure created downstream by a vacuum pump (or by a subject’s inhalation). A schematic representation of the PreciseInhale system is shown in Fig. 1.

Powder samples were loaded into the PreciseInhale dosing chamber in accurately weighed doses of approximately 1 mg (spray-dried AMP, spray-dried, and micronised adenosine) or 0.7 mg (micronised AMP and CPA). These quantities were found to generate the highest emitted fraction from the system with minimal risk of nozzle blockage. The system tracks the emitted dose during the measurement by means of a light scattering signal (Casella Microdust Pro, Casella CEL Inc., Buffalo, NY, USA), which is calibrated against the inhaled mass of the test substance [16]. As multiple doses are needed to obtain the full dose for an animal, differences in loaded dose have no implications for the administration.

The aerodynamic PSD of the aerosol was determined by cascade impaction analysis using the PreciseInhale system with a Marple cascade impactor (MSP Corp., Shoreview, MN, USA) attached to the exposure outlet by gravimetric measurement for micronised adenosine and micronised AMP only, at a flow rate of 2 L/min. A superimposed flow of 225 mL/min was applied to the pneumotachograph, which corresponds to the superimposed flow needed to ensure that fresh aerosol is presented to the animals during the in vivo exposure. The remaining 1775 mL/min airflow was drawn in through the air bypass on the impactor (see Fig. 1). The fractions available for bronchial, alveolar and total lung deposition were calculated using Multiple-Path Particle Dosimetry Model software (ARA, USA) and a breathing simulation model for the guinea pig (tidal volume 1.7 mL and 60 breaths/min). Measurements were performed in triplicate. For each replicate measurement, loading of multiple consecutive doses was required to obtain the full dose (ten for adenosine, five for AMP).

### Bronchial challenge in allergic guinea pigs

#### Animals

Male Dunkin–Hartley guinea pigs (400–450 g; Harlan, UK) were used throughout this study. Guinea pigs were housed on-site for at least 7 days prior to experimentation and given free access to food and water. They were maintained in cages containing bedding and enrichment with a 12-h day/night cycle. All studies were carried out under the UK Animals (Scientific Procedures) Act of 1986.

#### Passive sensitisation

The procedure for passive sensitisation of the guinea pigs has been described previously [14], although a lower dose of ovalbumin was administered in this study with a similar level of sensitisation. Ovalbumin was dissolved in saline (5 mg/mL) and mixed with aluminium hydroxide solution (*v*/*v* 1:10). This solution was injected intraperitoneally (i.p.) into recipients (1 mg/kg) on day 0 and again on day 10. On day 17, blood was collected via a cannula inserted into the carotid artery under i.p. anaesthesia with urethane (1.75 g/kg) and added to heparin (0.2 mL; 100 U/mL). Blood was centrifuged (2000×*g* for 15 min) and the plasma collected and stored at − 20 °C until further use. For passive sensitisation, this anti-ovalbumin guinea pig plasma was injected intravenously (i.v.) via the saphenous vein of recipient naïve conscious guinea pigs (1 mL per animal). Passively sensitised guinea pigs received an ovalbumin challenge (10 mg/mL for 1 h) 1 day prior to airway responsiveness assessment on days 7–10 (post injection).

#### Anaesthetic protocol

**Fig. 2:**
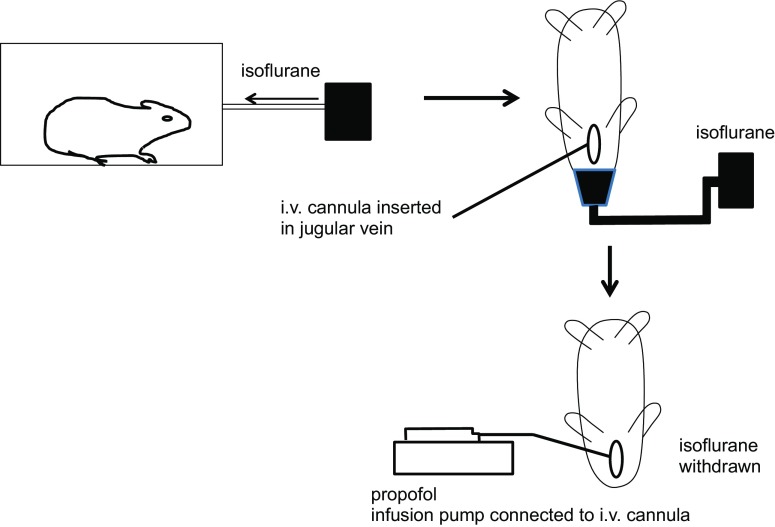
Schematic presentation of the anaesthetic protocol for spontaneously breathing, intratracheally cannulated guinea pigs

Guinea pigs were placed in an induction chamber and exposed to isoflurane (5%; 2 L/min). Once laterally incumbent, animals were removed from the chamber and isoflurane continued to be administered via a facemask. Adequate anaesthesia was achieved when guinea pigs no longer responded to a toe pinch. Isoflurane was then reduced to 3% (2 L/min). A midline incision was subsequently made in the neck of the guinea pig. The jugular vein was cannulated for i.v. administration of propofol. Isoflurane was then withdrawn and 100 μL propofol was slowly infused over 3 min, after which it was continuously infused (3 mL/h) using an infusion pump. The carotid artery was cannulated for heart rate and blood pressure measurements. The trachea was cannulated for attachment to the PreciseInhale system and ventilator. In Fig. 2, a schematic overview of the new anaesthetic protocol is given.

#### Dose calculations

The highest nebulised dose was based on previous studies using 10 mg/mL AMP [14], which equals to 7.7 mg/mL adenosine in terms of molarity. The highest dry powder doses of 215 μg adenosine and 280 μg AMP were calculated to correspond to the highest nebulised doses based on nebuliser output rate (0.34 mL/min), inhalation time (10 s) and inspiratory fraction of the respiratory cycle (0.50). The highest i.v. doses (0.77 mg/kg adenosine and 1 mg/kg AMP) were based on preliminary studies that established an increase in resistance of approximately 100% above baseline. Three dose levels (10, 50 and 100% of highest dose) were administered per delivery method.

#### Measurement of airway responsiveness

**Fig. 3:**
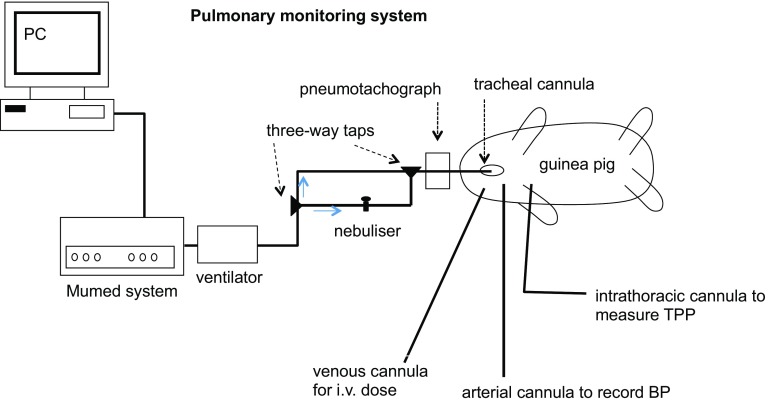
Schematic presentation of the integrated experimental setup for ventilation, nebulised aerosol administration and measurement of the pulmonary function of the guinea pigs. BP blood pressure, TPP transpulmonary pressure

In total, 18 groups of animals (two compounds × three delivery methods × three doses) were tested, with three to four animals in each test group. Six groups of animals were exposed to dry powder aerosols of micronised adenosine or micronised AMP via the PreciseInhale system. The tracheal cannula was connected to the outlet of the aerosol chamber (see Fig. 1), and animals were monitored for 2–3 min before aerosol exposure to ensure stable spontaneous breathing. After exposure, these animals were transferred to a heated mat and the cannula was then attached to the ventilator and pneumotachograph to record lung function parameters (see Fig. 3). The animals were artificially ventilated at a rate of 60 breaths/min and 1 mL air/100 g body weight. Six groups were exposed to adenosine or AMP via nebulised aerosol (Aeroneb, Aerogen, USA), and six further groups received adenosine or AMP i.v., all of these whilst on the ventilator at the same ventilation rate and tidal volume. For the nebulisation procedure, 1 mL of solution was added to the nebuliser. Once activated, the ventilator flow was turned (using a three-way tap) to pass through the nebuliser, which then forced the nebulised solution into the lungs for 10 s. The nebuliser was switched off and the ventilator flow changed back to flow directly to the animal (Fig. 3). Total lung resistance (R_L_), dynamic lung compliance (C_dyn_) and mean arterial blood pressure were recorded before and after adenosine receptor agonist administration using a pneumotachograph and pressure transducer (Validyne Engineering, USA) and automated lung function recording system (Pulmonary Monitoring System, version 9.2; Mumed, UK) as described previously [14].

#### Data analysis

Airway obstruction was calculated as % increase in R_L_ above baseline and % decrease in C_dyn_ below baseline, for which the individual animal’s own baseline values were used. Data are expressed as mean ± standard error of the mean (SEM). Due to the exploratory nature of the study, no power analysis was performed to determine the group size. Rather, three to four animals per group were tested to ensure sufficient data per group for observing trends, in case any of the groups contained a non-responding animal. No formal statistical analysis was performed either.

## Results

### Dry powder characterisation

The primary PSDs of spray-dried (adenosine and AMP) and micronised (adenosine, AMP and CPA) powders are shown in Table 1. All powders largely consisted of particles in the respirable size range (< 5 μm), and both spray-dried powders and the three micronised powders had very similar size distributions.

**Table 1:** Primary particle size distributions of the adenosine receptor agonist formulations

	X_10_ (μm)	X_50_ (μm)	X_90_ (μm)	FPF_< 5 μm_ (%)
*Spray-dried*				
Adenosine	1.03	2.19	3.97	96.7
AMP	0.83	2.25	4.84	91.2
*Micronised*				
Adenosine	0.66	1.42	2.93	100
AMP	0.65	1.40	3.38	100
CPA	0.66	1.42	3.03	100

X_10_, X_50_ and X_90_ represent the 10, 50 and 90% values from the cumulative volume undersize curve; the X_50_ value equals the volume median diameter (VMD). Data obtained with RODOS dispersion at 3 bar. *n* = 2, only the mean is shown because the variation is very low

*AMP* adenosine 5′-monophosphate, *CPA* 6 *N*-cyclopentyladenosine, *FPF*_*< 5 μm*_ fine particle fraction <5 μm as percentage of the delivered dose

**Fig. 4:**
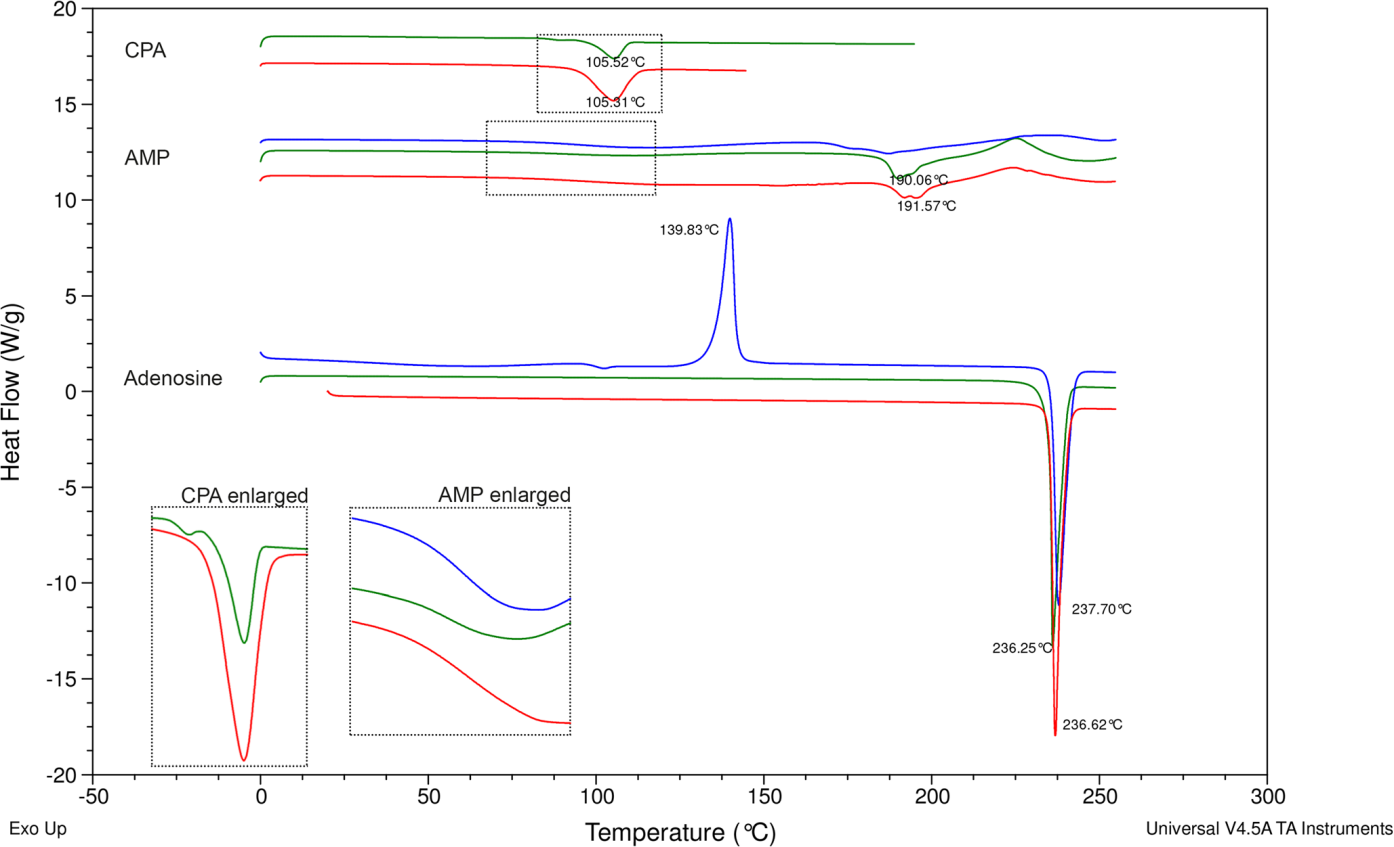
Differential scanning calorimetry thermograms of adenosine, adenosine 5′-monophosphate (AMP) and *N*^6^-cyclopentyladenosine (CPA) starting materials (red) and after micronisation (green) or spray drying (blue). Offset on the y-axis has been used for presentation purposes and clarity

DSC thermograms (heat flow versus temperature) of the adenosine receptor agonists as received and after spray drying or micronisation (Fig. 4) indicated that all starting materials showed one endothermal peak corresponding to the melting temperature of that compound (adenosine 236 °C, AMP 191 °C, CPA 105 °C), which was followed by degradation (exothermal event) in the case of AMP. AMP furthermore exhibited a step in the baseline (change in heat capacity) at 96 °C, which is considered to be a transition of amorphous content from glass into rubbery state. The thermograms of micronised adenosine and AMP were comparable to their starting materials. However, micronised CPA exhibited an additional thermal event at 87 °C, suggesting a glass transition. The energy that is expended during the endothermal event (melting) at 105 °C is lower compared to the starting material, confirming that the crystalline content of the powder is reduced. Spray-dried adenosine and spray-dried AMP also exhibited a transitory thermal event at 100 and 96 °C, respectively. Amorphous adenosine content then underwent crystallisation (exothermal event) at 140 °C and subsequent melting (endothermal event) at 237 °C. For spray-dried AMP, the endothermal melting peak was not present, indicating this powder was also (mostly) amorphous.

XRPD analysis confirmed that both adenosine and CPA as received were fully crystalline; AMP as received and micronised CPA were partly amorphous, partly crystalline and spray-dried adenosine and spray-dried AMP were fully amorphous (data not shown).

### Dry powder dispersion with the PreciseInhale system

**Fig. 5:**
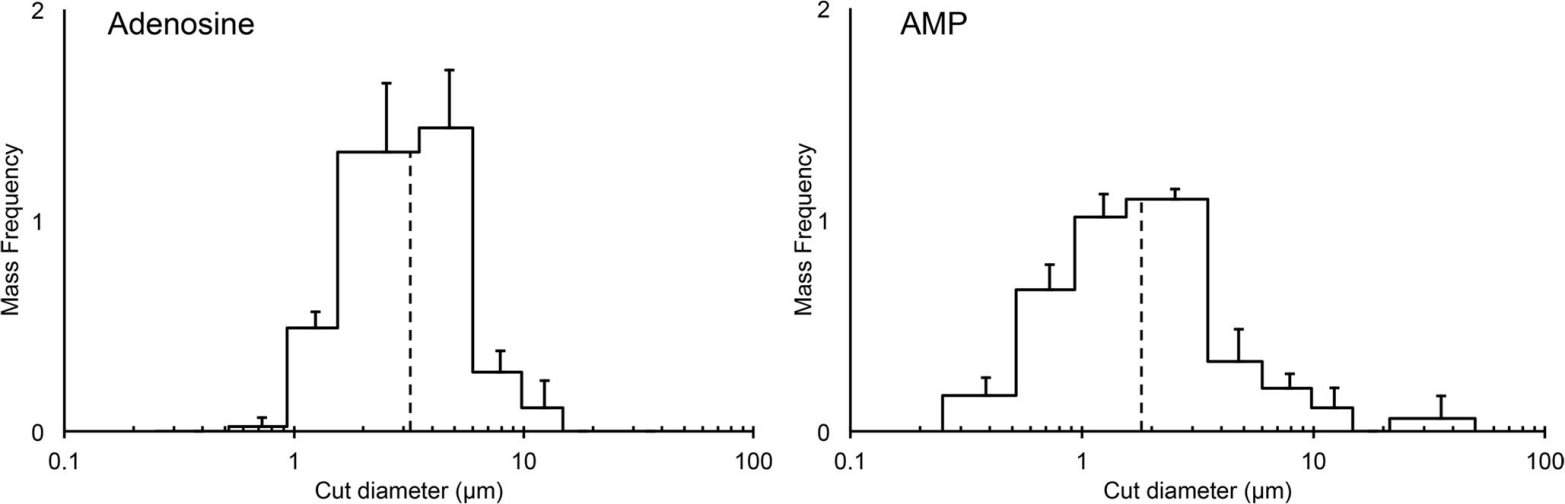
Aerodynamic particle size distribution of the delivered dose of adenosine (left) and adenosine 5′-monophosphate (AMP; right) generated with the PreciseInhale system obtained with cascade impaction analysis. The dotted line indicates the mass median aerodynamic diameter (MMAD). *n* = 3, mean ± SD shown

Five adenosine receptor agonist formulations were tested in the PreciseInhale system. For three formulations, spray-dried adenosine, spray-dried AMP and micronised CPA, an emitted fraction < 1% was obtained. This was due to the formation of hard agglomerates in the loading chamber of the PreciseInhale system retaining over 90% of the loaded dose. Micronised adenosine and micronised AMP were successfully dispersed with emitted fractions of 10 and 27% respectively (Table 2). Although micronised adenosine and AMP had very similar primary PSDs, with a volume median diameter (VMD) of 1.42 and 1.40 μm, respectively (Table 1), the aerodynamic PSD in the aerosol clouds from the PreciseInhale system were different (Fig. 5), with AMP exhibiting a mass median aerodynamic diameter (MMAD) of 1.8 μm and adenosine an MMAD of 3.2 μm. Since adenosine had a lower emitted fraction, this difference in PSD may be due to the loss of the finest fraction (< 1 μm) in the aerosol chamber by diffusive deposition on the wall of the chamber. Since very fine particles are also likely to be exhaled, the total lung deposition fraction was calculated to be higher for adenosine than for AMP, which was reflected in the target doses to be administered to obtain the desired deposited dose (452 μg for adenosine and 900 μg for AMP) (Table 2).

**Table 2:** Aerosol characteristics and performance parameters of dry powder dispersion with the PreciseInhale system used to calculate the target top dose of the micronised adenosine and micronised AMP formulations for administration to guinea pigs using the PreciseInhale system

	Emitted fraction (%)	MMAD (μm)	GSD	Total lung deposition fraction (%)	Ratio bronchial/alveolar deposition	Desired deposited dose (μg)	System target dose (μg)
Adenosine	10	3.2	1.8	48	70:30	215	452
AMP	27	1.8	2.2	31	63:37	280	900

Emitted fraction and MMAD (and GSD) were obtained from the PreciseInhale system. Total lung deposition fraction and the ratio bronchial/alveolar deposition were calculated using Multiple-Path Particle Dosimetry Model software (ARA, USA)

*AMP* adenosine 5′-monophosphate, *GSD* geometric standard deviation, *MMAD* median mass aerodynamic diameter

### Bronchial challenge in vivo

**Fig. 6:**
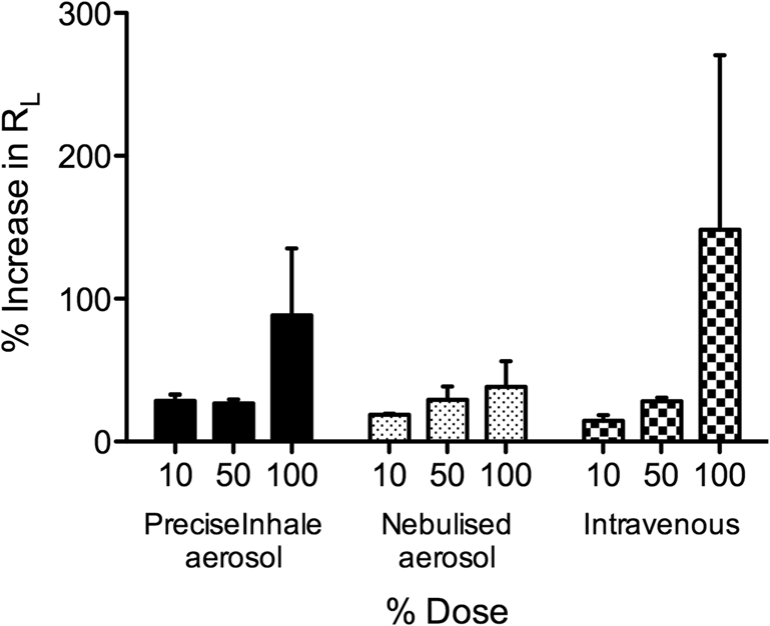
Dose-dependent responses in total lung resistance (R_L_) to adenosine administered via PreciseInhale dry powder aerosol, nebulised aerosol or i.v. injection. *n* = 3–4, mean ± SEM shown

**Fig. 7:**
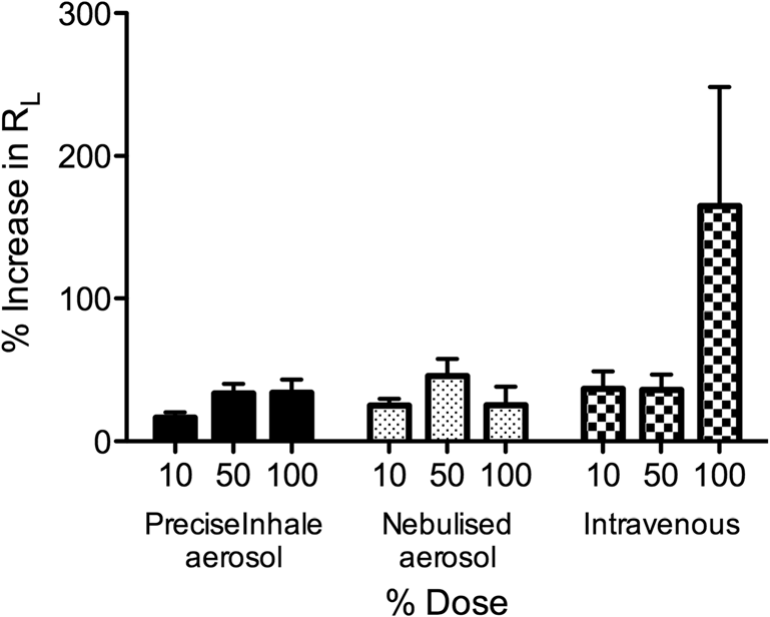
Dose-dependent responses in total lung resistance (R_L_) to adenosine 5′-monophosphate (AMP) administered via PreciseInhale dry powder aerosol, nebulised aerosol or i.v. injection. *n* = 3–4, mean ± SEM shown

Administration of both adenosine (Fig. 6) and AMP (Fig. 7) caused a dose-dependent increase in airway obstruction (R_L_) in sensitised guinea pigs when administered by each of the delivery methods. Obstruction of the lower airways (expressed in C_dyn_) exhibited a corresponding dose-dependent decrease in all groups (data not shown). The differences in the magnitude of responses produced by the three administration methods reflected differences in dose; the largest dose was delivered by i.v. (e.g., a guinea pig weighing 400 g received 308 μg adenosine and 400 μg AMP), followed by dry powder aerosol (215 μg adenosine and 280 μg AMP), and the dose delivered by nebulisation was presumably the lowest. Based on the values reported by the manufacturer (MMAD 2.1 μm, GSD 2.2 [18]), the deposited fraction from the nebulised aerosol is estimated to be 35%, which corresponds to doses of 75 μg adenosine and 98 μg AMP. Although the dry powder dose was based on the estimated dose delivered by nebulisation, the latter was not corrected for nebulised droplet size or exhaled fraction prior to experimentation, factors that the PreciseInhale system does take into account in calculating the (much higher) target dose. Thus, adenosine delivered using the PreciseInhale system produced a greater level of airway obstruction compared to the maximum dose administered via nebulisation. I.v. administration of adenosine achieved the greatest level of airway obstruction, although this route of administration is limited in practice due to greater systemic effects as adenosine receptor agonists cause a significant decrease in blood pressure, an effect minimised by selecting a route of administration targeting the adenosine directly to the lungs [14].

## Discussion

In this study, a pre-clinical model for studying airway responsiveness was developed based on aerosol administration of dry powder formulations of adenosine receptor agonists to spontaneously breathing, unconscious guinea pigs. To achieve this using the PreciseInhale technique required the development of a novel anaesthetic regime for guinea pigs, which was robust and allowed the animals to spontaneously breathe for the duration of the experiment.

For delivery of dry powder aerosols, the PreciseInhale system effectively dispersed micronised formulations of adenosine and AMP. However, it failed to disperse spray-dried formulations of these compounds or a micronised formulation of another adenosine receptor agonist, CPA. This was likely due to crystallisation of amorphous content (in the presence of a small percentage of residual water) in the powder upon applying the high-pressure pulse (100 bar) [19], which was used to disperse the powder into an aerosol cloud in the PreciseInhale system. During crystallisation, solid-state bridges were formed, resulting in hard agglomerates that were too large to be dispersed through the nozzle into the aerosol chamber. Reducing the pressure to 70 bar resulted in a slightly higher emitted fraction for micronised CPA, providing evidence for this crystallisation hypothesis. However, the emitted fraction for CPA was still below 1% and powder retention in the loading chamber was over 90%. Therefore, this compound in the formulation used was considered inappropriate for use with the current system. Formulation modifications such as the addition of stabilising excipients (e.g., sugars) or dispersion enhancers (e.g., leucine or magnesium stearate) could be explored to increase the emitted fraction. Additionally, system modifications could be made to produce powder aerosols using lower pressures where physical stability to high pressure is an issue. It would also be useful to develop a module to deliver liquid (nebuliser) aerosols to enable direct comparisons of different aerosol formulations using accurately controlled doses via a common delivery platform.

The micronised adenosine and AMP formulations were dispersed by the PreciseInhale system with no noticeable changes to the (minute) powder residues in the loading chamber of the system. Nevertheless, the emitted fractions were 10 and 27% for adenosine and AMP respectively. For relatively inexpensive compounds like adenosine and AMP, low emitted fractions do not necessarily pose a problem, as long as the results are reproducible. However, for more expensive compounds, low emitted fractions may be unacceptable, especially considering the further losses that occur during administration of the aerosols to animals. The system produces an aerosol that is presented to the animal continuously. However, since the animal breathes spontaneously in this setup, only during inspiration does the aerosol actually deposit in the lungs, and of those aerosolised particles that are available for deposition in the lungs, yet another fraction will be exhaled. Consequently, the amount of powder needed to be fed into the system was larger still, with 10–15 mg adenosine needed to obtain the desired deposited dose of 215 μg per animal (effective deposition 1–2% of loaded dose) and 7–10 mg AMP for a deposited dose of 280 μg (effective deposition 3–4% of loaded dose).

Respirable aerosol exposure in small animals is a great challenge. Intratracheal delivery via instillation of liquid sprays or powders deliver drug to the lungs with less losses compared to nasal or intratracheal inhalation of respirable aerosols, but at the expense of uneven distribution of the test material in the lungs [20–22]. With respiratory aerosol exposures supplying fresh aerosols to avoid rebreathing of aerosol, there is a theoretical maximum delivered dose of 50% based on the inhalation-exhalation breathing cycle [23], which converts to an upper limit of ~ 25% in rodents using intratracheal inhalation when the deposition fraction is applied. However, this often decreases to below 10% if typical losses to equipment deposition are included.

Although the system delivers a dose of aerosol “efficiently” to an individual animal, there were issues with the time required to deliver the dose and interfacing the apparatus for aerosol delivery with that for measuring lung function. This may have been the reason why the dose-dependent increase that was observed with adenosine could not be seen with AMP. AMP was less effective at causing airway obstruction compared with adenosine and indeed administration as a powder did not achieve a significant level of airway obstruction. This may be due to the longer time (> 12 min) required for delivery of the full dose, which could have been so long that it partly overlapped with the onset and maxima of the compound’s bronchoconstrictive effects. As the animals had to be transferred from the PreciseInhale system to the ventilator for the lung function measurement, it is likely that the maximum effect was missed for the highest AMP doses. One of the drawbacks of the current system is the inability to monitor lung function simultaneously with administering compounds through the PreciseInhale system to intratracheally cannulated or intubated animals, although the manufacturer of the system has indicated that they are working on integrating lung function measurements into the equipment (personal communication). Nonetheless, the current setup does allow for bronchoactive compounds (e.g., bronchodilators) to be administered directly to the lungs to determine the time course of the bronchoprotective effect when the spasmogen is subsequently administered by nebulisation whilst the animal is on the ventilator. Furthermore, there is scope for nose-only administration through the PreciseInhale system, which may allow for studying chronic exposure of powders to animals, as surgery would not be required.

The system’s advanced feedback system, based on measurement of light scattering at the outlet of the aerosol chamber, enables improved control of the dose that is administered to the individual animals. The key advantage of this feedback system is that it leads to reduction of the variation of delivered dose per animal [20], which is especially important in toxicological and pharmacokinetic studies. Reduced variability in delivered dose should also lead to less variability in effect, but the group sizes in this proof-of-concept study were too small to formally assess whether a reduction in variation was obtained. Additionally, bronchoconstriction may not be the best outcome measure to evaluate variability because of large inter-individual variation in airway responsiveness.

In conclusion, we have confirmed that inhalation of adenosine and AMP caused airway obstruction in spontaneously breathing, unconscious, ovalbumin-sensitised guinea pigs, whether these compounds are administered by solution or dry powder aerosol into the lungs. The PreciseInhale system accurately administered micronised powder aerosols directly to the lungs of individual guinea pigs, although some powders were not physically stable due to high-pressure dispersion.
